# Linear Phase Sharp Transition BPF to Detect Noninvasive Maternal and Fetal Heart Rate

**DOI:** 10.1155/2018/5485728

**Published:** 2018-03-29

**Authors:** Niyan Marchon, Gourish Naik, K. R. Pai

**Affiliations:** ^1^Padre Conceicao College of Engineering, Goa, India; ^2^Goa University, Goa, India

## Abstract

Fetal heart rate (FHR) detection can be monitored using either direct fetal scalp electrode recording (invasive) or by indirect noninvasive technique. Weeks before delivery, the invasive method poses a risk factor to the fetus, while the latter provides accurate fetal ECG (FECG) information which can help diagnose fetal's well-being. Our technique employs variable order linear phase sharp transition (LPST) FIR band-pass filter which shows improved stopband attenuation at higher filter orders. The fetal frequency fiduciary edges form the band edges of the filter characterized by varying amounts of overlap of maternal ECG (MECG) spectrum. The one with the minimum maternal spectrum overlap was found to be optimum with no power line interference and maximum fetal heart beats being detected. The improved filtering is reflected in the enhancement of the performance of the fetal QRS detector (FQRS). The improvement has also occurred in fetal heart rate obtained using our algorithm which is in close agreement with the true reference (i.e., invasive fetal scalp ECG). The performance parameters of the FQRS detector such as sensitivity (Se), positive predictive value (PPV), and accuracy (F_1_) were found to improve even for lower filter order. The same technique was extended to evaluate maternal QRS detector (MQRS) and found to yield satisfactory maternal heart rate (MHR) results.

## 1. Introduction

All over the world, approximately 2.65 million stillbirths occur during pregnancy or labour especially in developing countries giving rise to the need for effective monitoring techniques with regard to fetal health [1]. FHR monitoring is important to recognize pathologic conditions, typically asphyxia, with sufficient warning so as to enable intervention by the clinician [2]. It is a screening modulus of the fetus to detect problems in advance that could result in irreversible neurological damage, even fetal death [3]. More than 85 percent of all live births in the United States undergo electronic fetal monitoring [4]. Indeed, fetal health monitoring has a significant importance in obstetrical procedures and is now widely accepted as the need of the hour.

With electronic fetal monitoring (EFM), the following expectations came: provision of accurate FECG information, information of value in diagnosing fetal distress, prevention of fetal death or morbidity, and superiority over many methods. The fetus can be monitored electronically by two methods: direct and indirect. In the direct invasive method, the FHR is measured by a scalp electrode which is attached to the fetal scalp by means of a coiled electrode [5]. In the indirect electronic monitoring method, such as using ultrasound Doppler principle with uterine contractions, FHR can be monitored but not as precisely as the direct invasive FECG [2]. However, the invasive procedure has a risk of infection to the fetus. The ultrasound transducer with the coupling gel is applied to the mother's abdomen where fetal heart response is best detected. During this measurement, the pulsations of the maternal aorta could be detected and erroneously considered as FHR [6]. The noninvasive FECG (NIFECG) by indirect method can therefore be used to overcome all these limitations by placing the surface electrodes such as the 12 lead ECG electrodes over the maternal abdomen [7]. The maternal thoracic ECG can also be taken as a reference signal along with the abdominal ECG (aECG). A study was conducted during labour of about 75 pregnant mothers, to check the accuracy and reliability of the NIFECG [8]. It was found that the NIFECG recordings were more accurate than the conventional external methods in comparison with the direct fetal scalp recordings. Therefore with EFM, using NIFECG recordings is the most suitable for long-term ambulatory use [9].

### 1.1. Fetal Physiology

Fetal distress and fetal asphyxia are too broad and vague to be applied with any precision to clinical situations. Uncertainty regarding the diagnosis based on interpretation of FHR patterns has given rise to reassuring and nonreassuring patterns [6]. Reassuring FHR patterns include the normal baseline FHR, moderate accelerations, and variability with fetal movement assuring the well-being of the fetus, whereas nonreassuring FHR patterns include tachycardia (FHR baseline more than 160 bpm), bradycardia (FHR baseline is less than 110 bpm) [10], prolonged decelerations, and so on. The severe and prolonged hypoxia induces a prolonged fall in FHR [11]. Some causes of fetal bradycardia include congenital heart block [12]. The baseline fetal heart rate is greater than 160 bpm. It is reported that the fetal body movements affect variability [13], while the baseline variability increases with advancing gestation [14]. It is also reported that reduced variability with decelerations is associated with fetal acidemia [15]. An FHR who has a consistently flat baseline with no variability and without decelerations within the normal baseline rate limit range may reflect a neurological damage in the fetus [16]. It is important that we understand the parameters of the fetal ECG signal which further aids the analysis of the fetal status and the EFM, during pregnancy or labour [1].

### 1.2. Previous Methods to Extract NIFECG from aECG

Researchers in the biomedical field in the areas of fetal extraction and fetal analysis have done extensive work in the last two decades. A large number of detection and extraction techniques are used to separate the FECG from the maternal ECG. Independent Component Analysis (ICA) is a statistical technique, and its accuracy is based on using a large number of noise-free maternal abdominal input channels. For ICA to function correctly, certain conditions such as (i) the number of measured signals should be equal to or greater than the number of input sources, (ii) it should possess an instantaneous linear time invariant mixing matrix, and (iii) the input sources should be statistically independent [17]. In our application, the first two do not fully satisfy because the artifacts increase the number of sources and fetal movement leads to a noninvariant mixing matrix [18]. ANFIS is an adaptive noise cancellation system which requires an additional maternal thoracic ECG signal as reference signal for adaptive cancellation of the maternal ECG. This method depends on how well one trains the ANFIS structure to compute the estimated output FECG signal [19, 20]. Subtraction method is a simple technique, but the major challenge is that the amplitude of the thoracic MECG rarely matches the scale of the MECG present in the aECG signal [21]. As a result, correct FECG is hardly ever obtained. Wavelet transform method can be used for preprocessing stage to suppress noise, and maternal cancellation can be done by template subtraction [22]. Correlation techniques are not very efficient and effective in the detection of nonstationary signals like ECG [23]. As IIR filtering being a nonlinear method [24], our technique of using linear phase sharp transition FIR filter is less complex and does not involve many iterations as the filter response is specified precisely over the entire band. With the knowledge of the fiduciary edges and the fair estimate of the spectral overlap of maternal and fetal ECG, accurate FHR and maternal heart rate can be obtained. Our technique being single channel lead makes it very convenient and comfortable for a maternal home care for long-term monitoring.

The amplitude of MECG is at least 10 times larger than that of FECG, and the signal-to-noise ratio (SNR) of the MECG is less than unity [25]. The separation of these two ECG signals becomes even more complex as the maternal and fetal ECG overlap both in time and frequency domain [3]. The aECG signal is further affected with the low frequency noise of 0.5 Hz [26] due to baseline wandering where the amplitude of the ECG signal also varies by about 15% with respiration [2]. The other noise which affect the aECG are 50/60 Hz power line interference (PLI) [27], electromyographic noise in the uterus and the muscles of the abdomen, and other motion artefacts [28].

### 1.3. Linear Phase Sharp Transition Band-Pass Filters

The location of the passband and its width are critical factors that affect the design implementation of the filter. Usually sharp transition BPF are realised by the composite filters of high-pass and low-pass filters as done in [29]. The interpolated FIR technique was used wherein, every time the centre frequency of the BPF was changed, the two composite filters had to be redesigned. Approximate expressions for the value of interpolating factor and filter hardware required are derived which minimizes the total arithmetic hardware used which is the overall band-pass realization. The two-branch structure realization is more efficient than the conventional direct form realization with an increase in the number of delays [30]. Another technique of symmetric BPF is given in [31]. The filter is implemented by two parallel, quadrature filter branches with each branch derived from a complex modulation of a low-pass-interpolated FIR filter by complex exponentials. The input signal is modulated with a sine/cosine sequence in order to achieve the desired frequency shift in the frequency response.

In the current work, we propose a two-stage method to obtain noninvasive FQRS from a single lead maternal abdominal signal by first applying the designated fiduciary edges to the linear phase sharp transition (LPST) FIR band-pass filter with a sharp transition width. In the second stage, an FQRS detector is used based on Pan Tomkins QRS detector algorithm [32]. The QRS detector module consists of an amplitude squarer, moving window integrator, moving average filter, and an adaptive threshold process which effectively detects the fetal R-peaks.

## 2. Methodology Using LPST FIR Band-Pass Filter

Our proposed technique of integrated LPST FIR band-pass filter has low computational complexity. Normally, the composite low-pass and high-pass filters are to be redesigned for any change in the centre frequency and pass band width for the desired BPF. Our proposed technique for the integrated BPF design departs from this approach completely. It eliminates the need for a centre frequency and the fixed passband width as it is used in [33]. Our design of LPST FIR BPF allows the user to set the cutoff frequencies for a narrow pass band width. It also incorporates a very linear sharp transition width while reducing the effects due to Gibb's phenomenon and thereby reducing the passband ripple of the filter [34].

### 2.1. LPST FIR BPF Model and Design

**Figure 1:**
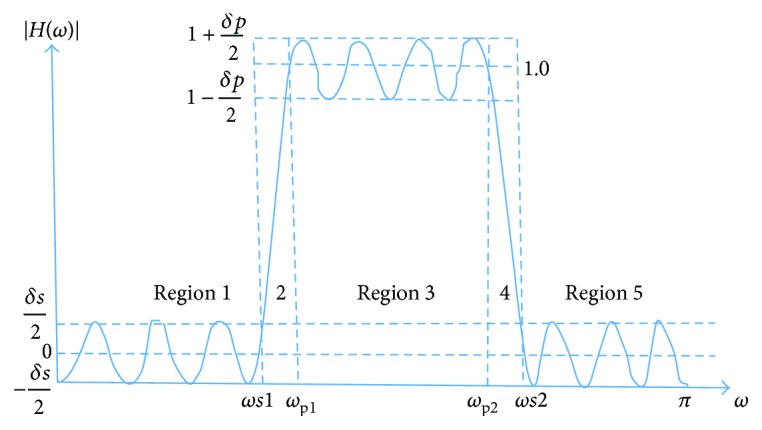
Magnitude response *H*(*ω*) of the band-pass filter.

In this section, the design of a LPST FIR BPF is presented. For the proposed filter model, the five regions of the filter response are modelled using trigonometric functions of frequency. The filter model magnitude response *H*(*ω*) is shown in Figure 1.

The frequency responses for the five regions are listed as follows:

(1) $$\begin{array}{c} \left.\begin{array}{l} Region\;1:H\left(\omega \right)=-\frac{\delta_{s}}{2}\cos\left(k_{1}\omega \right)\;0\leq \omega \leq \omega_{s1},\\ Region\;2:H\left(\omega \right)=k_{2}\left(\omega -\omega_{s1}\right)\;\omega_{s1}\leq \omega \leq \omega_{p1},\\ Region\;3:H\left(\omega \right)=1+\frac{\delta_{p}}{2}\sin\left(k_{3}\left(\omega -\omega_{p1}\right)\right)\;\omega_{p1}\leq \omega \leq \omega_{p2},\\ Region\;4:H\left(\omega \right)=1-k_{4}\left(\omega -\omega_{p2}\right)\;\omega_{p2}\leq \omega \leq \omega_{s2},\\ Region\;5:H\left(\omega \right)=-\frac{\delta_{s}}{2}\sin\left(k_{5}\left(\omega -\omega_{s2}\right)\right)\;\omega_{s2}\leq \omega \leq \pi . \end{array}\right\} \end{array}$$

Using (1), the filter design parameters *k*_1_, *k*_2,_*k*_3,_*k*_4_, and *k*_5_ for the five regions of the band-pass filter are evaluated and listed as follows:

(2) $$\begin{array}{c} \;\left.\begin{array}{l} k_{1}=\frac{2\pi m_{1}+\left(\pi /2\right)}{\omega_{s1}}\\ k_{2}=\frac{1}{\left(\omega_{p1}-\omega_{s1}\right)},\\ k_{3}=\frac{\left(2m_{3}+1\right)\pi }{\left(\omega_{p2}-\omega_{p1}\right)},\\ k_{4}=\frac{1}{\left(\omega_{s2}-\omega_{p2}\right)},\\ k_{5}=\frac{2\pi m_{5}+\left(\pi /2\right)}{\left(\pi -\omega_{s2}\right)\;}, \end{array}\right\} \end{array}$$

where *ω*_s1_ and *ω*_s2_ are the stopband edge frequencies while *ω*_p1_ and *ω*_p2_ are the passband edge frequencies. *δ*_s_ and *δ*_p_ are the stopband attenuation and passband ripple, respectively, while *m*_1_, *m*_3_, and *m*_5_ are integers.

The impulse response coefficients *h*(*n*) for the FIR band-pass filter are obtained from [35]

(3) $$\begin{array}{c} h\left(n\right)=\frac{1}{\pi }\left[\int_{0}^{\pi }H\left(\omega \right)\sin\left(k\omega \right)d\omega \right]. \end{array}$$

Substituting the magnitude response *H*(*ω*) for each region from (1) and (2) in (3), we get

(4) $$\begin{array}{c} h\left(n\right)=\left\{\left(\frac{\delta_{s}}{4\pi }\right)\left[\frac{\cos\left(\left(k+k_{1}\right)\omega_{s1}\right)-1}{\left(k+k_{1}\right)}+\frac{\cos\left(\left(k-k_{1}\right)\omega_{s1}\right)-1}{\left(k-k_{1}\right)}\right]\right\}+\left\{\left(\frac{k_{2}}{k\pi }\right)\right.\left[\left(-\omega_{p1}\right)\cos\left(k\omega_{p1}\right)+\left(\omega_{s1}\right)\cos\left(k\omega_{s1}\right)\right]-\left(\frac{k_{2}}{k^{2}\pi }\right)\left[\sin\left(k\omega_{p1}\right)-\sin\left(k\omega_{s1}\right)\right]+\left(\frac{k_{2}\omega_{s1}}{k\pi }\right)\left[\cos\left(k\omega_{p1}\right)-\cos\left(k\omega_{s1}\right)\right]+\left(-\frac{1}{\pi }\right)\left[\frac{\cos\left(k\omega_{p2}\right)-\cos\left(k\omega_{p1}\right)}{k}\right]+\left(\frac{\delta_{p}}{4\pi \left(k-k_{3}\right)}\right)\left[\sin\left[\left(k-k_{3}\right)\omega_{p2}+k_{3}\omega_{p1}\right]-\sin\left(k\omega_{p1}\right)\right]+\left(\frac{-\delta_{p}}{4\pi \left(k+k_{3}\right)}\right)\left[\sin\left[\left(k+k_{3}\right)\omega_{p2}-k_{3}\omega_{p1}\right]-\sin\left(k\omega_{p1}\right)\right]+\left(\frac{1}{k\pi }\right)\left[-\cos\left(k\omega_{s2}\right)+\cos\left(k\omega_{p2}\right)\right]+\left(\frac{k_{4}}{k\pi }\right)\left[\left(\omega_{s2}\right)\cos\left(k\omega_{s2}\right)-\left(\omega_{p2}\right)\cos\left(k\omega_{p2}\right)\right]+\left(\frac{k_{4}}{k^{2}\pi }\right)\left[\sin\left(k\omega_{s2}\right)-\sin\left(k\omega_{p2}\right)\right]+\left(\frac{k_{4}\omega_{p2}}{k\pi }\right)\left[-\cos\left(k\omega_{s2}\right)+\cos\left(k\omega_{p2}\right)\right]+\left\{\left(\frac{-\delta_{s}}{4\pi }\right)\left[\left(\frac{\sin\left(\left(k_{5}-k\right)\pi -k_{5}\omega_{s2}\right)+\sin\left(k\omega_{s2}\right)}{\left(k_{5}-k\right)}\right)-\left(\frac{\sin\left(\left(k_{5}+k\right)\pi -k_{5}\omega_{s2}\right)-\sin\left(k\omega_{s2}\right)}{\left(k_{5}+k\right)}\right)\;\right]\right\},\;\text{where }k=\left[\left(\frac{N-1}{2}\right)-n\right]. \end{array}$$

Equation (4) is the expression for the band-pass filter model impulse response *h*(*n*). We can choose the effective pass band width (*ω*_p2_ ~ *ω*_p1_) such that (*ω*_s1_ ~ *ω*_p1_) = (*ω*_s2_ ~ *ω*_p2_), as small as possible for sharp transition of passband edge. Once *ω*_p1_, *ω*_p2_, *ω*_s1_, and *ω*_s2_ are chosen, *k*_1_, *k*_2_, *k*_3_, *k*_4_, and *k*_5_ are determined.

### 2.2. Expression for Frequency Response Coefficients of a LPST FIR Filter

Let *h*(*n*) given by (4) be the impulse response coefficients of an *N* point linear phase FIR filter [36] where 0 ≤ *n* ≤ *N* − *1* and

(5) $$\begin{array}{c} k=\left[\left(\frac{N-1}{2}\right)-n\right],\;n=0,1,2,\ldots ,\left(\frac{N-3}{2}\right),\text{for }N\;odd \end{array}$$

and

(6) $$\begin{array}{c} k=\left[\left(\frac{N-1}{2}\right)-n\right],\;n=0,1,2,\ldots ,\left(\frac{N}{2}-1\right),\text{for }N\;even. \end{array}$$

In the case of antisymmetric response with *N* odd [37], the frequency response of the FIR band-pass filter is given as

(7) $$\begin{array}{c} Hr\left(\omega \right)=2\overset{\left(N-3\right)/2}{\underset{n=0}{\sum }}h\left(n\right)\sin\left(\omega \left(\frac{N-1}{2}-n\right)\right). \end{array}$$

This response is most suitable for the proposed band-pass filter as *H*(0) = 0 and *H*(*π*) = 0. If we refer to (5), *k* is an integer for *N* odd. Other constraints are as follows: (i) In (2), *k* ≠ *k*_1_, *k* ≠ *k*_3_, and *k* ≠ *k*_5_ and (ii) *k*_1_, *k*_3_, and *k*_5_ should not be integers. However, *k*_2_ and *k*_4_ do not have any constraints.

### 2.3. Fetal Frequency Spectrum

In our experiment, to extract the QRS of the MECG and FECG from online Physionet databases [38], we used (i) Abdominal and Direct Fetal Electrocardiogram Database (adfecgdb) which provides abdominal ECG recordings (channels 2 to 5) for 5 minutes each from five different subjects during the 38–41-week gestation period [39, 40]. In addition, for each subject, a simultaneously recorded scalp or direct fetal ECG record (channel 1) is a golden reference in the evaluations to be made on the respective records. (ii) The Non-Invasive Fetal Electrocardiogram Database (nifecgdb) provides 55 records of different lengths from a single subject taken from the 20th week of pregnancy [41]. Channels 1 and 2 represent maternal thoracic ECG signals while channels 3 to 6 are abdominal ECG recordings with only MQRS reference annotations. The Q-R-S fiducial edges of the thoracic MQRS and the invasive FQRS signals were obtained for each record. The fast Fourier transform (FFT) was obtained for the above records, an average frequency range for MQRS was found to be 10–34 Hz while the average FQRS spectrum was 20–56 Hz.

FHR varies with gestation age, ranges from 70 beats per minute (bpm) at four weeks to 175 bpm at 12 weeks and further decreases to a range of 110 to 160 bpm at full term [42]. The FECG bandwidth ranges from 0.05 to 100 Hz [2] with an average value of 140 bpm. However, in comparison, the maternal bpm normally ranges from 50 to 210 bpm with an average of 80 or 89 bpm [42].

We assumed the maternal beats per minute range to be 70–100 bpm (1.166_min_–1.666_max_ bps) and the fetal beats per minute range to be 110–140 bpm (1.833_min_–2.333_max_ bps). The minimum and maximum fetal-to-maternal (f/m) frequency ratios are obtained to compute the average f/m frequency ratio from (8) and (9).

(8) $$\begin{array}{c} Minimum\;\frac{f}{m}\;frequency\;ratio=\left(\frac{{fetal}_{bps}}{{maternal}_{bps}}\right)\min=\frac{1.833}{1.166}=1.572, \end{array}$$

(9) $$\begin{array}{c} Maximum\;\frac{f}{m}\;frequency\;ratio=\left(\frac{{fetal}_{bps}}{{maternal}_{bps}}\right)\max=\frac{2.333}{1.666}=1.400, \end{array}$$

(10) $$\begin{array}{c} Average\;\frac{f}{m}\;frequency\;ratio=\left(\frac{1.572+1.4}{2}\right)=1.486. \end{array}$$

We selected the frequency spectrum for MQRS to be 18 to 35 Hz [42] to estimate the lower and higher fetal QRS fiduciary edges from (10) as

(11) $$\begin{array}{c} {FQRS}_{lower\;fiduciary\;edge}={MQRS}_{lower\;fiduciary\;edge}\times average\;\frac{f}{m}\;frequency\;ratio=18\times 1.486\sim 27\;Hz,\\ {FQRS}_{upper\;fiduciary\;edge}={MQRS}_{upper\;fiduciary\;edge}\times average\;\frac{f}{m}\;frequency\;ratio=32\times 1.486\sim 48\;Hz. \end{array}$$

From (11), we get the lower fiduciary edge of FQRS to be of 27 Hz which will remove all the low frequency noise including baseline wander frequencies and upper fiduciary edge of 48 Hz which will remove the 50 Hz and its PLI harmonics along with the high frequency noise [27]. The fetal QRS frequency band spectrum can also be further narrowed down so as to avoid the frequency band overlap of MECG and FECG. Accordingly, the upper fiduciary edge of the fetal QRS spectrum is chosen to be 49 Hz or 98*π* rad/sec. The lower fiduciary edge of the fetal spectrum is set to 35 Hz or 70*π* rad/sec, since the upper MQRS edge is reported to be approximately 35 Hz [42]. This results in a fetal pass band width of 14 Hz or 28*π* rad/sec.

### 2.4. FQRS Detector

To obtain the FQRS from the band-pass filtered signal, we tried looking at various algorithms including the peak-finding logic using the Hilbert transform [43]. We proposed a simple QRS detection algorithm which is based on the Pan Tomkins algorithm [32]. The modified FQRS detector comprises of four stages: (i) amplitude squarer, (ii) moving window integrator, (iii) moving average filter, and (iv) adaptive threshold. The filtered FECG signal from the LPST FIR BPF is given to the amplitude squaring stage wherein the signal is squared point by point. This nonlinear process enables the high frequency fetal R-peak signals to be further enlarged and minimizes the other lower frequency components. Further, we used a moving window integrator with a sampling frequency (fs) of 1 KHz. This integrator effectively summed the area under the squared waveform over a fixed window interval, advanced one sample interval at a time. The width of the moving window was set to 75 sample interval for FQRS detection while a window of 152 samples wide was adjusted for MQRS detection. A too large window can merge the QRS-integrated waveform and T wave, where as if the window is too narrow, a QRS complex could produce several peaks at the output stage [32]. Additionally, a moving average filter was also used which smoothened the integrated signal and compute a single fetal R-peak. Based on the algorithm in [44], an adaptive threshold is automatically generated to adjust to float above the unwanted noise peaks. Initially, the signal peak value is adjusted manually as per the amplitude of each record [44]. The fetal R-R interval (Δ*n*) is calculated as (*n*_*i*+1_ − *n*_*i*_) where *n_i_* is the time index corresponding to the *i*th computed fetal R-peak at the output of the FQRS detector (*i* = 1, 2… where *i* is an integer). The FHR is computed for each record using

(12) $$\begin{array}{c} FHR\left(bpm\right)=\frac{\left(fs\times 60\right)}{\Delta n}. \end{array}$$

## 3. Results

### 3.1. Synthesis of the LPST FIR Band-Pass Filters

The LPST FIR band-pass filter was implemented using (7). The following FQRS band-pass fiduciary edge cutoff frequencies (rad/sec) were substituted as per Figure 1: *ω*_s1_ = 70*π*, *ω*_p1_ = 72*π*, *ω*_p2_ = 96*π*, and *ω*_s2_ = 98*π*. Also stop band and passband ripple *δ*_s_ = *δ*_p_ = 0.01. Equal transition width at both ends was chosen for the pass band to be 2*π* rad/sec or 1 Hz. The measurement of the magnitude response of the band-pass filters is compared in Tables 1 and 2 along with the filter design specifications.

### 3.2. Performance Analysis of the FQRS Detector

**Figure 2:**
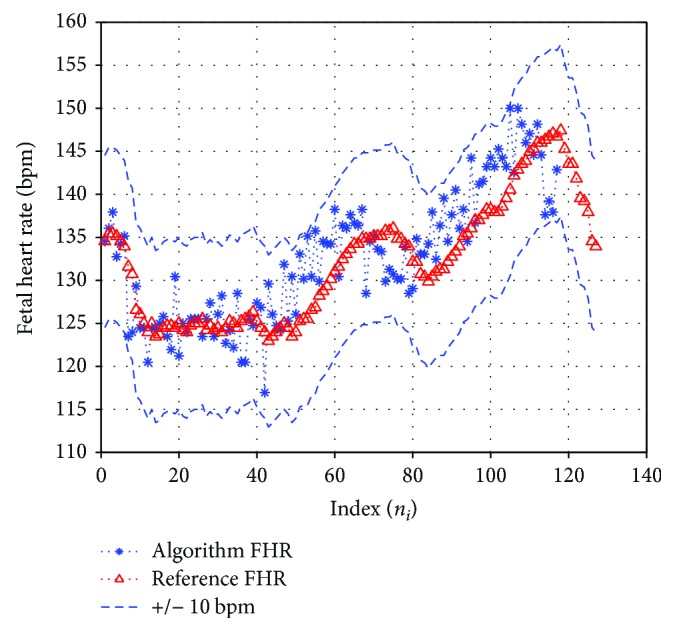
Illustration of the true reference FHR (direct scalp ECG) plotted with our algorithm computed FHRV for record r08 of adfecgdb (channel four) for one-minute trace. Blue dotted lines indicate ±10 bpm tolerance with respect to the reference FHR trace.

As per the guidelines of ANSI/AAMI (ANSI/AAMI/ISO EC57 1998/(R) 2008) [1, 45], the following classical statistics for evaluating QRS detectors were used to evaluate the FQRS detector. Sensitivity (Se), positive predictive value (PPV), and accuracy (F_1_) are shown in (13) where TP, FN, and FP are true positive (correctly identified fetal R-peaks), false negative (missed fetal R-peaks), and false positive (falsely identified R-peaks), respectively. The test points assumed here are to be within ±10 bpm of their corresponding reference measurement. The true reference, namely, scalp fetal R-peak annotations from each record of the Physionet database, was compared with our experimental measured values which was implemented using Matlab toolbox. For example, we evaluated our algorithm for the adfecgdb database for the one-minute record (r08) of channel 4 and found the TP = 132, FN = 1, and FP = 0. The sensitivity, PPV, and F1 were obtained to be 99.24%, 100%, and 99.61%, respectively. The average FHR values for the true reference and algorithm FHR were computed to be 132.09 bpm and 132.59 bpm, respectively.  Figure 2 illustrates the true reference FHR bpm plotted with our algorithm-based fetal heart rate variability (FHRV) for record r08. The dotted lines indicate the ±10 bpm tolerance assumed in our case with respect to the true reference FHR trace. It was seen that the difference between the reference FHR and algorithm FHR for most R-peaks was less than the ±8 bpm.

(13) $$\begin{array}{c} Se=\frac{TP}{TP+FN},\\ PPV=\frac{TP}{TP+FP},\\ F_{1}=2\left(\frac{Se\cdot PPV}{Se+PPV}\right). \end{array}$$

## 4. Discussions

**Figure 3:**
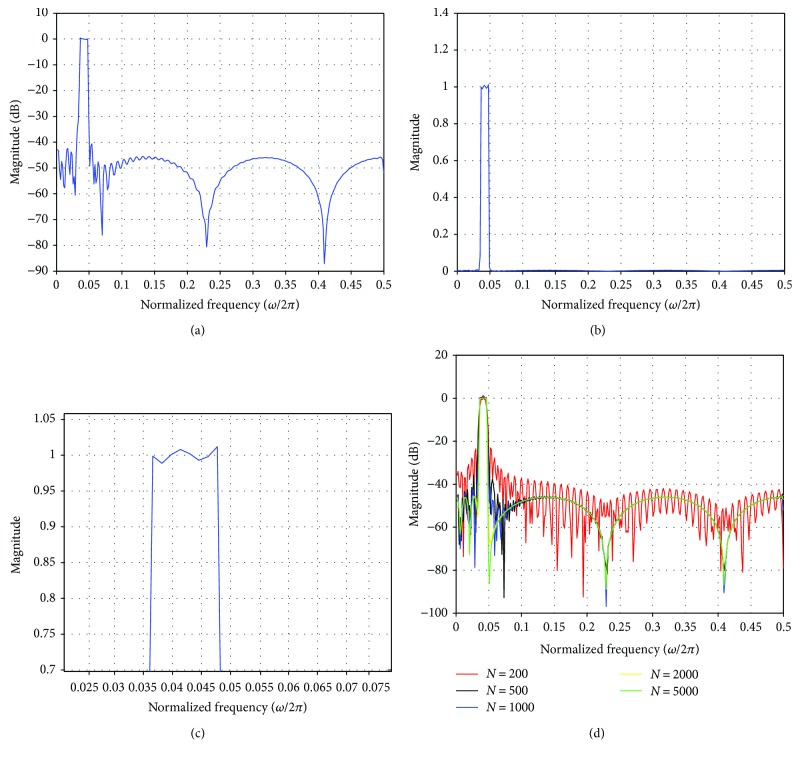
(a) Magnitude response of the proposed BPF with filter order *N* = 1001. (b) Linear plot. (c) Magnified view of the passband. (d) Magnitude response of the BPF LPST for various filter orders (*N*).

We designed a LPST FIR band-pass filter such that the magnitude *H*(*ω*) in the passband and stopband are not constant but inserted a small amount of ripple of 0.01 in the stopband as well as passband so that Paley-Wiener criterion is not violated [46]. The FIR filter was designed for sharp transition width (*ω*_s_ − *ω*_c_) of 1 Hz or 2*π* rad/sec. The magnitude responses of the proposed band-pass filter are shown in Figures 3(a)–3(c).  Table 3 depicts the performance of the filter for various filter orders (*N*). There is a reduction of Gibb's phenomenon with these filter designs. For conventional FIR sharp transition filters, the peak passband ripple due to Gibb's phenomenon is about 18% [34, 46]. In our proposed LPST FIR band-pass filters, the passband losses are quite low as can be seen from Table 2. It can also be seen that the stopband attenuation surpasses the design specification at higher orders and the passband ripple decreases for higher filter order as seen from Table 3. The sampling rate *N* = 1001 is much higher than the Nyquist rate (approximately 200 Hz) and is chosen to improve the quality of the extracted FECG. Various filter orders (*N* = 201, 501, 1001, 2001, and 5001) were implemented to check the performance of the filters as shown in Figure 3(d). These filters are unlike the classical filters in that they possess a narrow stopband and/or passband and also sharp transition regions. The magnitude response, the linear plot, and the magnified view of the BPF are shown in Figures 3(a)–3(c), respectively, with the filter order *N* equal to 1001.

**Figure 4:**
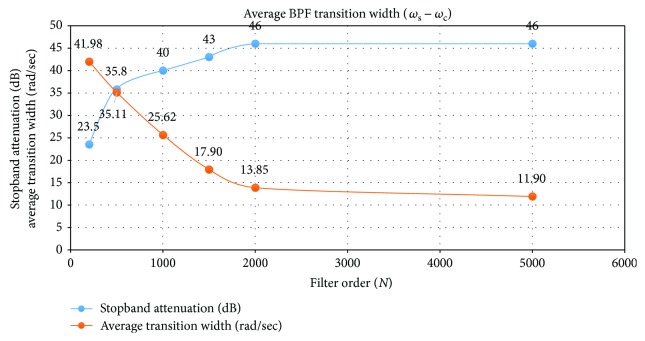
Average BPF transition width and stopband attenuation for various filter order (*N*).

**Figure 5:**
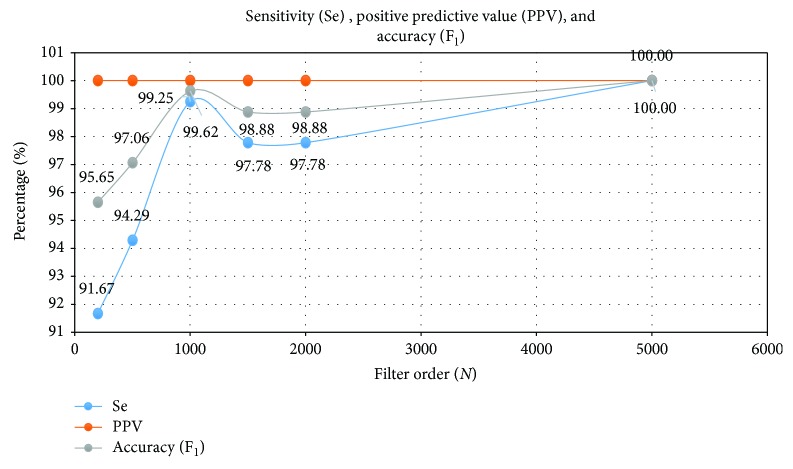
Sensitivity, positive predictive value, and accuracy of record r08 (adfecgdb) with TP = 132 for various filter order (*N*).

As seen from Figure 4, the average transition width approaches the design specifications at higher orders. The performance curves of Se, PPV, and F_1_ are highly linear in the range of filter orders (*N*) from 2001 to 5001 as seen in Figure 5. This improvement may be due to better filtering at higher order.

**Figure 6:**
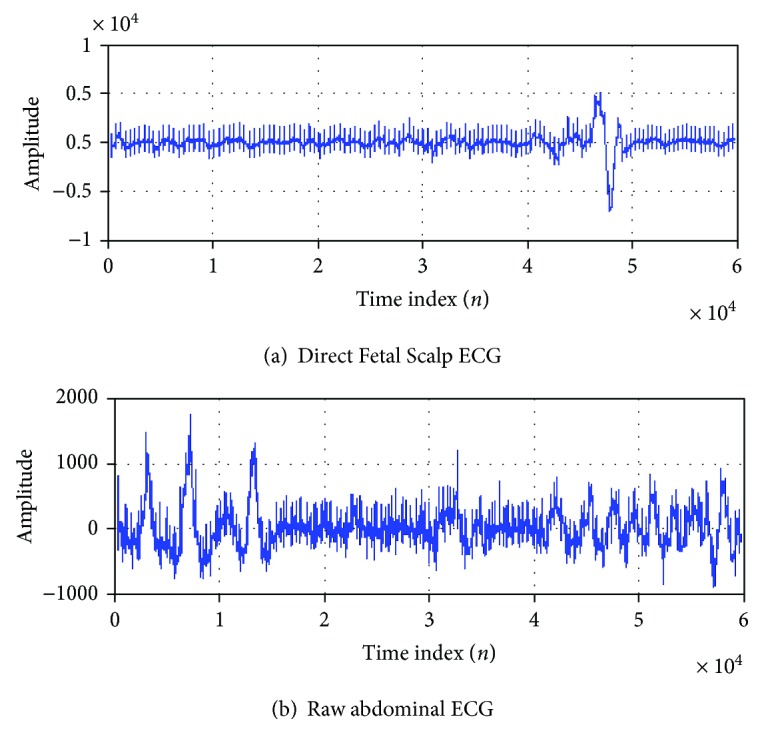
(a) Direct fetal scalp ECG signal (channel one) as standard reference FECG. (b) Raw maternal aECG of adfecgdb database (channel 4—record r08).

**Figure 7:**
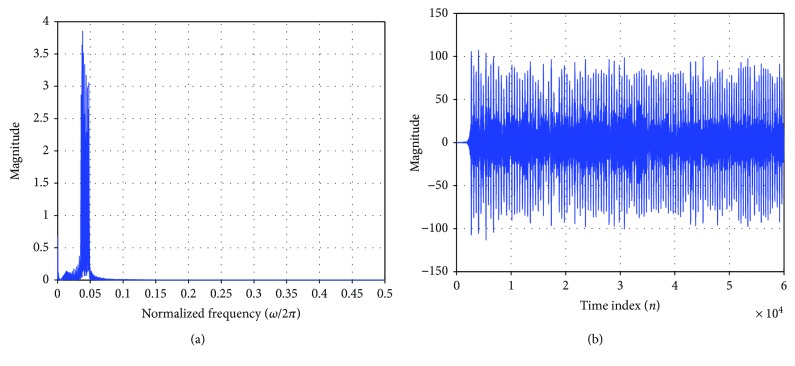
(a) Frequency spectrum of the narrow sharp transition band-pass filtered signal. (b) FQRS time domain signal after band-pass filtering.

The direct fetal scalp ECG is the standard reference FECG signal (channel one) as shown in Figure 6(a). The raw maternal aECG signals were taken from channel 4—record r08 of the adfecgdb database as shown in Figure 6(b). The frequency spectrum of the signal which passed through BPF filters (frequencies between 35 Hz and 49 Hz) is shown in Figure 7(a). The band-pass filtering effectively gives us the required frequency spectrum of the FECG, which can be seen in the time domain plot in Figure 7(b).

**Figure 8:**
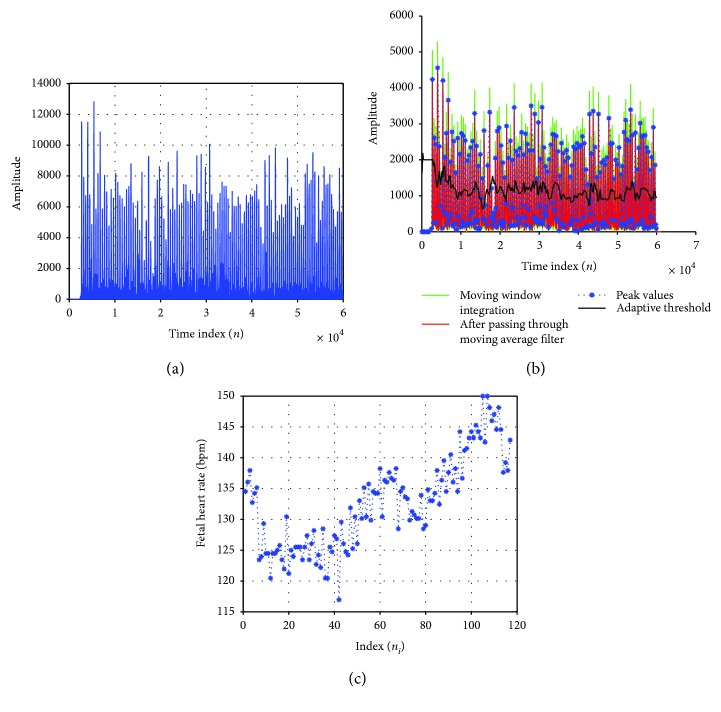
Noninvasive FHR detector for *N* = 5001 for adfecgdb database (channel 4—record r08). (a) Amplitude squaring of fetal R-peaks. (b) Moving window integration and adaptive threshold. (c) Fetal heart rate variability.

When FQRS signal is passed through an amplitude squarer, the predefined positive peaks are prominently amplified as shown in Figure 8(a).  Figure 8(b) shows the moving window integrator which integrates this signal with a selected window size, effectively picking the correct fetal R-peak indices. An illustration from Figure 8(b) shows that the time indices (*n*) for the first two detected fetal R-peaks are 3155 and 2709, respectively, which are above the adaptive threshold value. As shown in Figure 8(c), the FHR at these *n*_*i*=1_ and *n*_*i*=2_ are computed to be 134.52 bpm using (12).

**Figure 9:**
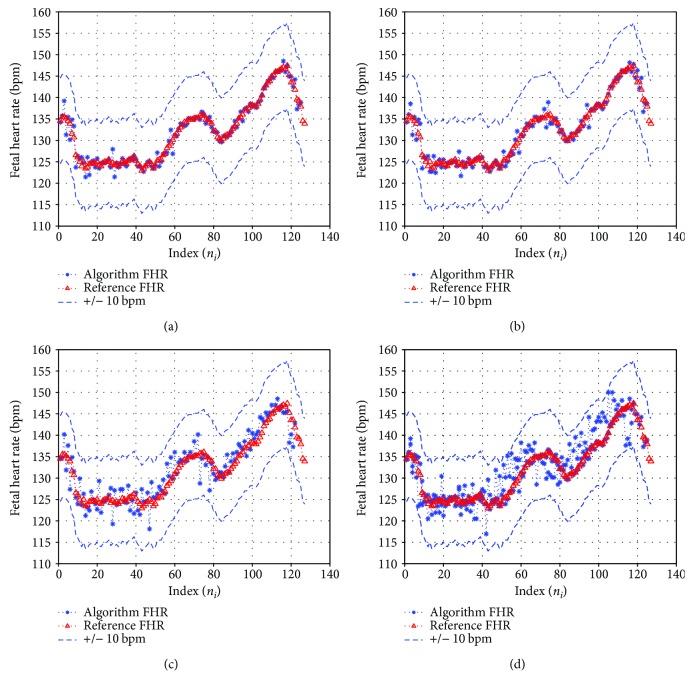
Illustration of the true reference FHR plotted with our algorithm computed FHRV for four sets of fetal frequency fiduciary edges of the BPF. The signal used is a one-minute trace of record r08, channel 4 of adfecgdb with filter order *N* = 5001 (*n_i_* is the time index corresponding to the *i*th computed fetal R-peak at the output of the FQRS detector). (a) Type 1: 27 Hz–53 Hz. (b) Type 2: 27 Hz–48 Hz. (c) Type 3: 35 Hz–53 Hz. (d) Type 4: 35 Hz–48 Hz. The dotted lines indicate the ±10 bpm tolerance assumed with respect to the reference FHR trace.

Among the four types of fetal frequency fiduciary edges of the BPF, type 1 (27 Hz*–*53 Hz) will absorb some of the PLI in the ECG record, whereas type 2 (27 Hz*–*48 Hz) avoids PLI unlike type 1 but has a partial overlap spectrum of maternal ECG. Similarly, type 3 (35 Hz*–*53 Hz) will again have PLI problem but has no maternal spectrum overlap. Finally, the type 4 (35 Hz to 48 Hz) can be considered optimum since the maternal spectrum overlap and PLI are absent. In spite of narrowing the spectrum in this case, there are no missing fetal beats. The illustration of the true reference FHR plotted with our algorithm computed FHR for the four sets of fetal frequency fiduciary edges of the BPF is shown in Figure 9.

**Figure 10:**
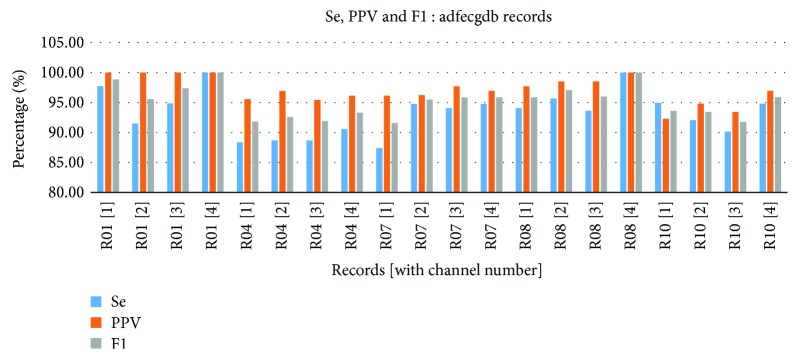
Sensitivity, positive predictive value, and accuracy for all the four channels of the 5 adfecgdb records.

The FQRS detection performance parameters, Se, PPV, and F_1_, were calculated for all the four channels for each of the 5 adfecgdb records using the type 4 fetal frequency fiduciary edges as shown in Figure 10. It was observed that all the above three parameters were 100% for channel 4 of records r01 and r08. The missed fetal R-peaks (FN) were seen in some channels of records r04, r07, and r10, while the falsely identified fetal R-peaks (FP) were the least in most of the records.

**Figure 11:**
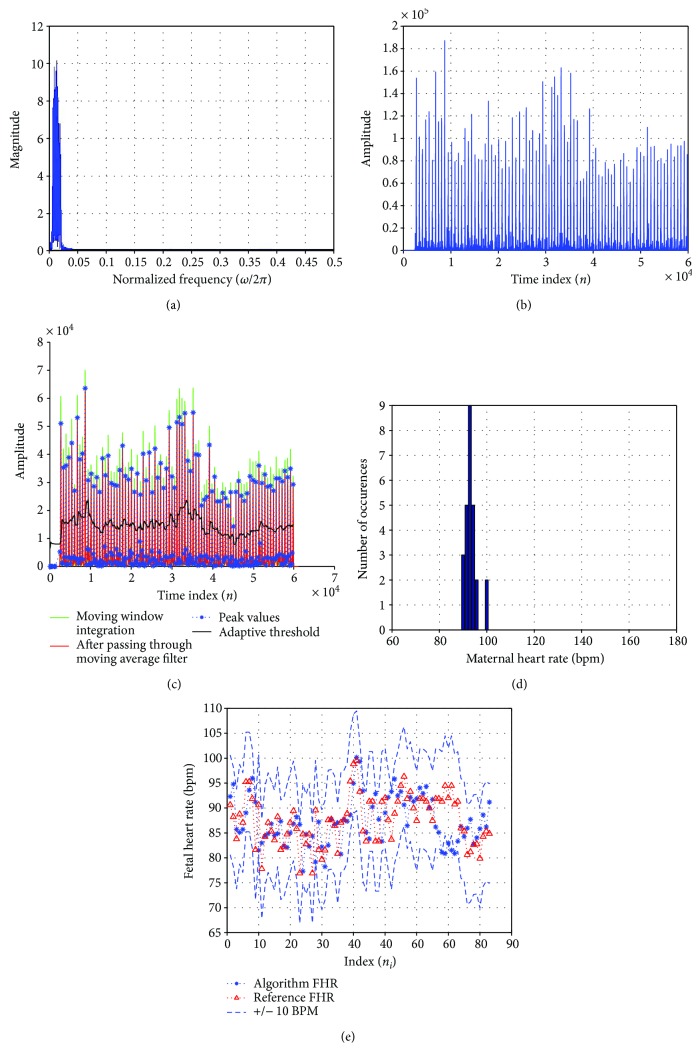
Using LPST BPF for MHR detection with fiduciary edges, *ω*_s1_ = 10*π* and *ω*_s2_ = 40*π* for record r01 (channel 3) of adfecgdb. (a) Frequency spectrum of the narrow BPF signal. (b) Amplitude squaring of maternal R-peaks. (c) Moving window integration and adaptive threshold. (d) Histogram of the MHR. (e) The true reference MHR plotted with our algorithm computed MHR.

**Figure 12:**
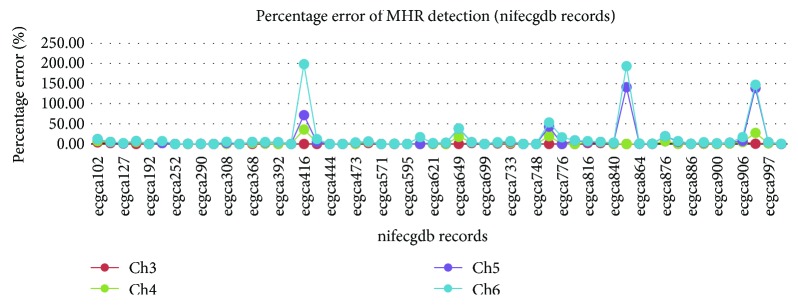
Percentage error of MHR detection compared with the MHR references for all the four channels of the 55 nifecgdb records.

It is found that this technique can be extended to detect maternal heart rate merely by changing the fiduciary edges of the BPF to *ω*_s1_ = 10*π* and *ω*_s2_ = 40*π* as shown in Figure 11. An illustration of the adfecgdb record r01 (channel 3) detected TP = 89, FN = 3, and FP = 0 to compute Se, PPV, and F_1_ to be 96.74%, 100%, and 98.34%, respectively, as shown in Figure 11(e). Similarly, the QRS detection algorithm was tested for the MHR using the Physionet nifecgdb database for all 55 records with 3 to 4 channels each. It was observed that the MHR for all the four aECG channels for most records closely matched the MQRS reference annotations. Abdominal signals from channels 5 and 6 of records such as ecgca416, ecgca597, ecgca649, ecgca771, ecgca848, and ecgca986 displayed a large percentage error difference of computed MHR bpm value as compared with the reference MHR due to the degradation of the acquired aECG signals as seen in Figure 12.

## 5. Conclusion

In this paper, we described a technique of fetal heart rate detection performed noninvasively. This technique was implemented using a linear phase sharp transition FIR band-pass filter. We considered four types of fetal frequency fiduciary edges characterized by varying amounts of overlap with maternal ECG spectrum. Type 4 was found to be optimum with no PLI, no maternal spectrum overlap, and no fetal beats missed. It is found that increasing the filter order has improved the average transition bandwidth, passband ripple, and stop band attenuation of the filter. The fetal R-peaks generated by our algorithm were compared with the scalp fetal R-peak annotations from the Physionet databases. The algorithm-generated fetal R-peaks were found to be in close agreement with each other including the average FHR values of the true reference and algorithm FHR. Similarly, other performance indices such as Se, PPV, and F_1_ were found to have promising results, even for lower filter orders. The same technique was successfully extended to maternal heart rate detection.

## Figures and Tables

**Table 1:** Band-pass LPST filter specifications of passband and stopband edges (type 4 fiduciary edges) along with measured magnitude response values.

Band-pass LPST filter (filter order (*N*) = 1001)	Stopband edge (*ω*_s1_) rad/s	Passband edge (*ω*_p1_) rad/s	Passband edge (*ω*_p2_) rad/s	Stopband edge (*ω*_s2_) rad/s
Design specifications	70*π*	72*π*	96*π*	98*π*
Measured specifications	64.68*π*	73.22*π*	92.3*π*	100.08*π*

**Table 2:** Band-pass LPST filter specifications of transition bandwidth, passband ripple, and stopband attenuation using type 4 fiduciary edges along with measured magnitude response values.

LPST filter (filter order (*N*) = 1001)	Transition bandwidth (*ω*_p1_–*ω*_s1_) rad/s	Transition bandwidth (*ω*_s2_–*ω*_p2_) rad/s	Max. passband loss (dB)	Min. stopband attenuation (dB)
Design specifications	2*π*	2*π*	±0.873	40
Measured specifications	8.54*π*	7.78*π*	+0.47, −0.13	40

**Table 3:** Variations of passband loss and stopband attenuation for BPF with various filter orders (*N*).

Filter order (*N*)	201	501	1001	1501	2001	5001
Passband loss (dB)	1.5	±0.5	±0.13	±0.1	±0.04	±0.03
Stopband attenuation (dB)	23.5	35.8	40.6	43	46	46
